# Developments on Constitutive Material Model for Architectural Soda-Lime Silicate (SLS) Glass and Evaluation of Key Modelling Parameters

**DOI:** 10.3390/ma16010397

**Published:** 2023-01-01

**Authors:** Andrzej Malewski, Marcin Kozłowski, Jacek Podwórny, Marcin Środa, Wojciech Sumelka

**Affiliations:** 1 Institute of Structural Analysis, Poznan University of Technology, 61-138 Poznan, Poland; 2 Department of Structural Engineering, Silesian University of Technology, 44-100 Gliwice, Poland; 3 Łukasiewicz Research Network, Institute of Ceramics and Building Materials, Refractory Materials Lab, 44-100 Gliwice, Poland; 4 Faculty of Material Science and Ceramics, AGH University of Science and Technology, 30-059 Kraków, Poland

**Keywords:** glass formation, soda-lime silicate glass, numerical modeling, modern architecture, literature review, glass production

## Abstract

Architectural soda-lime silicate glass (SLS) is increasingly taking on complex shapes that require more detailed numerical analysis. Glass modeling is a thoroughly described topic with validated constitutive models. However, these models require a number of precise material parameters for SLS glass, and these are very sensitive to changes in glass composition. The currently available information is based on SLS glass tested in the late 1990s. As a result, most current publications are based on the above data. The object of this work was to analyze the available sources and update the information on selected key parameters for modeling. Using the currently utilized SLS glass in construction, the coefficient of thermal expansion (CTE), glass transition temperature, and the Young’s modulus have been experimentally investigated. The updated material parameters will allow for more accurate modeling of the SLS glass currently used in construction, and in consequence will make the prototyping process for glass with complex geometries possible to be transferred from the production stage to the design stage, resulting in shorter production times.

## 1. Introduction

The presence of glass in the history of mankind dates back to nearly 7000 BC, when the first glass fragments were produced in Mesopotamia and ancient Egypt. Subsequently, mankind learned to control the process of glass production, and around the first century BC, blown glass technology was developed [1]. The process continued to improve, and new types of glass were created, including colored glass used extensively in Europe during the Middle Ages. Glass, because of its unique nature, is a subject for continuous research. The most recent and best-suited definition of glass was proposed by [2] and can be described as “Glass is a nonequilibrium, non-crystalline condensed state of matter that exhibits a glass transition. The structure of glasses is similar to that of their parent supercooled liquids (SCL), and they spontaneously relax toward the SCL state. Their ultimate fate, in the limit of infinite time, is to crystallise”. It is also worth expanding the definition of “noncrystalline”, which describes that material lacks a periodic atomic structure in the long range, which is characteristic of crystals [1]. If the glass is considered in terms of what compounds can form glass, a more precise division can be made. For most of the history of mankind, silica has been the main ingredient used to form glass, but it is not a prerequisite for creating such a material. In fact, it was only after the 1900s that mankind began to better understand the process of glass formation and became familiar with other types of glass such as borosilicate glass. Currently, the main glass used in construction is soda-lime silicate glass (SLS).

The focus of this paper is to gather available information regarding the numerical modeling of SLS glass, which material parameters are required for simulation and to present the results of experimental tests of a few key material parameters, which were carried out on SLS glass samples from two float manufacturers frequently used in the building industry nowadays.

## 2. Materials and Methods

The article is structured into two parts. The first part serves as an introduction to the subject of glass modeling and lists the most important publications that have contributed to the development of the subject. Furthermore, the validated model is indicated and its structure is described. At the end of this section, the key material parameters for this model are summarized. In the second part, the methodology for testing the key material parameters using SLS glass is presented. The results of the tests of these key parameters and a comparison of the information currently available are described.

### 2.1. Literature Study Methods

**Figure 1:**
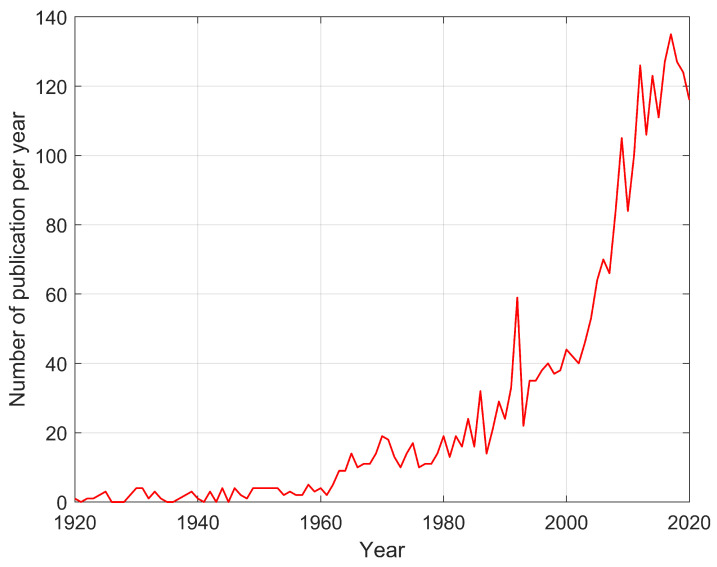
Number of publications released per year based on a selected combination of keywords.

The literature analysis first focused on understanding the history and current state of knowledge about mathematical models of glass developed since the beginning of interest in glass by the scientific community, i.e., the early 20th century. Attention has also been focused on numerical models and how these models have been implemented in computer calculation software. These assumptions were realized in the form of an analysis of numerous interrelated papers that provide the basis for current knowledge of glass modeling and a database of information on material parameters. The following keywords were selected for the publication search: soda, glass, viscoelastic, abaqus, Maxwell, WLF, slumping, Arrhenius, and relaxation. The Google Scholars database was then searched using Publish or Perish software [3] and results were collected from 250–500 items per keyword combination. As a result of the analysis, it was noticed that the keyword combination glass/viscoelastic/soda and glass/viscoelastic/soda/relaxation returns the highest number of results close to the topic of the publication. In total, a database of 2871 unique publications was created along with 3043 unique authors. Then, using Gephi software [4], the extracted results were visualized to find the interrelationships between them to find the key authors for the scope of the research and the publications with the greatest impact. Based on the collected data, the graph shown in Figure 1 was generated. As mentioned above, it can be observed that the last quarter century has seen a sharp increase in publications dedicated to the issue.

### 2.2. Experimental Study

As a result of the work on the first part, the focus was on measuring the coefficient of thermal expansion (CTE) by using TMA (Thermomechanical Analysis), glass transition temperature by using DSC (Differential Scanning Calorimetry) and changes in Young modulus at elevated temperatures by using RFDA (Resonant Frequency and Damping Analyser). Three samples each of annealed SLS float glass from two glass suppliers were tested. The first set of samples referred later as Sample A is 5 mm Eurowhite NG Float from glass manufacturer Euroglas. The second set of samples described later as Sample B is 5 mm UltraClear Float from glass manufacturer Guardian Glass. Both products comply with the composition requirements of the current standard EN 572-1 [5], which specifies magnitude of proportions by mass for individual constituents, i.e., silicone dioxide (SiO_2_) 69% to 74%, calcium oxide (CaO) 5% to 14%, sodium oxide (Na_2_O) 10% to 16%, magnesium oxide (MgO) 0% to 6%, aluminium oxide (Al_3_O_2_) 0% to 3% and others 0% to 5%.

CTE measurements were carried out using a TMA-7 Perkin-Elmer device working with Pyris software. Measurements were performed in the range from room temperature to the level at which the sample exhibits viscous flow. The three samples per supplier were heated at a rate of 10 K/min in a helium atmosphere at a contact force of 110 mN and a sensor with a base diameter of 3 mm. The values of the coefficient of linear expansion (CTE) were measured in the ranges up to 473 K, 573 K, 673 K and 773 K.

The second measurements (DSC) of the glass transition temperature were performed on a NETZSCH STA 449 F3 Jupiter thermal analyzer in platinum crucibles at a heating rate of 10 K/min in a synthetic air atmosphere with a flow rate of 40 mL/min up to 1273 K. Measurements were made on three samples per supplier for each type of glass. On the basis of the obtained curves, the transition temperature $T_{g}$ as a point on the DSC curve and the change of specific heat capacity accompanying the glass transition were determined.

The determination of the Young modulus and damping of the glass at a temperature range of 295 K to 873 K by means of RFDA (Resonant Frequency and Damping Analyser) produced by IMCE (Integrated Materials Control Engineering, Genk in Belgium) and equipped by HT 1700 furnace were carried out. The most important resonant frequencies are flexural, which are controlled by the Young’s modulus of the sample for isotropic materials. For predefined shapes such as rectangular bars, the dedicated software calculates the elastic properties of the sample using the dimensions, weight, and frequency of the sample according to ASTM E1876-15. The dimensions of the glass samples used were 6 × 35 × 150 mm^3^, the heating and cooling rate was 3 K/min. The samples were annealed at 873 K for 60 min.

## 3. Results

### 3.1. Literature Study

In history, many methods of glass production have been developed (crown glass, broad glass, sheet glass, plate glass), but it is the float process that has completely dominated glass production. Over time, it has become desirable for architectural glass to be as flat as possible. The breakthrough came in the 1950s when Sir Alastair Pilkington [6] developed the technology to produce floating glass. It made it possible to obtain perfectly flat glass without the need for lengthy grinding and polishing of the glass surface. Additionally, the entire production took place on an industrial scale, which made this process dominant in modern flat glass production.

As computers became more computationally capable and numerical methods developed, engineers began to create even more daring designs using glass as a building material. In general, this made it possible to simulate glass for a variety of issues ranging from the question of simulating glass destruction by an impacting object [7], the effect of temperature changes for insulated glass units [8] or simulating the effect of high temperatures for laminated glass [9]. In regards to curved glass, the last 25 years have seen a significant increase in interest in complex shapes and frequent nonlinear glass [10,11] and its architectural benefits. This is due both to the number of computer software that allows the design of such glazing [12] and the fact that technology has developed that has allowed one to reduce the unit cost of producing such glass. In the early days, the use of bent glass was mainly limited to the use of cold bent tempered glass, where the glass was bent to the substructure on site [13,14,15] or bent in an autoclave during the laminating process [16,17,18]. This was due to the fact that the production of such glass used flat float glass and was therefore relatively cheap. Hot-bent glass, on the other hand, is a much more complex and time-consuming production technology that requires a considerable amount of experience or the need for prototyping prior to serial production. For this reason, there is a need to be able to accurately simulate numerically the bending process in a furnace, which will reduce the number of trials needed and allow a preliminary selection of possible geometries.

The topic of simulating the behavior of glass at elevated temperatures is not new in the scientific world. There are a number of publications proposing various theoretical models, and even more publications that implement these theories into numerical models. Each of these theoretical models requires different parameters of the glass material to best reproduce the natural behavior of glass at elevated temperatures. However, a brief review of the literature clearly shows that there is a lack of research on material parameters for SLS glass currently used in construction.

**Figure 2:**
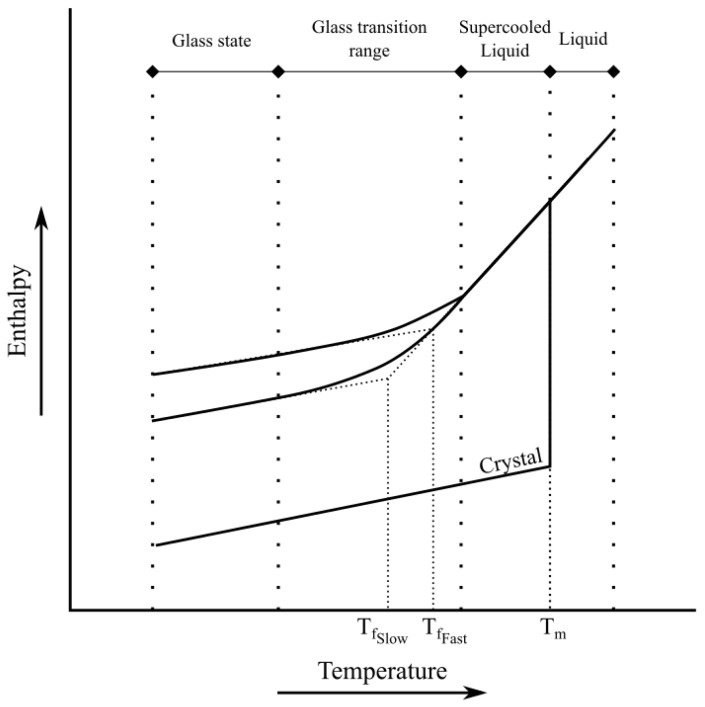
Glass transition phases.

As mentioned in the definition of glass, it exhibits a glass transition, and this process occurs within a specific temperature range called the glass transition range (see Figure 2). This creates three different states that all depended on the relevant temperature region, where the glass-forming material behaves as follows:

(1) Glass state ($T<T_{f}$);
(2) Supercooled liquid state ($T_{f}<T<T_{m}$);
(3) Liquid state ($T>T_{m}$).

where $T_{m}$ is the melting temperature, $T_{f}$ the fictive glass temperature. The $T_{f}$ is the conventional point of intersection of the extrapolated lines of the glass state (1) and the supercooled liquid state (2). This parameter can be described as an indicator of the deviation of the glass atomic structure from its equilibrium state. If the fictive temperature is equal to the current temperature, then the glass can be assumed to be in equilibrium [19]. It is not an exact method for describing the thermal history of glass, but it allows an approximate description of structural changes and its parameters during cooling. More on this is described in the paper. In addition, the concept of transformation temperature $T_{g}$ should be clarified further. As mentioned, this is a range of temperatures, and no single value can be determined unambiguously. Nevertheless, it is also considered convenient to determine the value on the basis of tests. The two methods described later (CTE and DSC measurements) make it possible to determine the value of the glass transition temperature $T_{g}$ on the basis of changes in heat transfer or thermal expansion, but because it depends on the method and the heating rate used, $T_{g}$ is not a constant material parameter of the glass.

The scope of interest is limited to the processing of the glass and thus the aforementioned range can be limited to glass and supercooled liquid states. All processes that directly affect the final product take place at the transition between these two states. Supercooled liquids are liquids that are cooled below their melting temperature of the crystal without crystallization [20,21]. Once the temperature drops further, the structure continues to rearrange, but there is no rapid decrease in enthalpy, as is happening for the phase transition. As can be noticed, glass that is cooled faster is transforming to the vitreous state more quickly and then will have a higher glass transition temperature when it starts to be heated. One of the only features of supercooled liquids is the massive increase in shear viscosity by a factor greater than $10^{14}$ [22,23]. At a certain point, the increase in viscosity becomes large enough that atoms cannot rearrange completely and equilibrium starts to run behind its equalized state, i.e., falling out of equilibrium [24,25,26]. This is why the glass transition phenomena are determined by a kinetic process. Considerable information on glassy and amorphous materials can be found in [27].

This unique behavior of glass makes it not easy to describe mathematically what happens in specific temperature ranges. The development of the current state of knowledge on how to model glass includes a couple of milestone publications that have contributed significantly to the advancement of this field. The first detailed study on various soda-lime glass compositions at that time was conducted by [28], where for the first time the changes in the viscosity of glass at high temperatures were studied in detail. However, as became apparent later, the main challenge was to describe the behavior of glass over a range of transition temperatures and to try to capture how stresses form in glass as it is cooled. The most important factor was to combine the mechanical response of the glass along with its thermal history and to describe the temperature and the time dependent stress relaxation.

As described by [29] the first model that appeared in 1948 [30] assumed an instantaneous change of glass from a liquid to a solid state as an elastic body when the glass transition temperature is reached. This assumption was a simplification, but allowed a relative determination of the residual stresses in the glass as a result of tempering. Subsequently, refs. [31,32] further developed Bartenev’s model in 1954 and 1961, respectively.

The next breakthrough was the publication by [33] in 1965, which assumed that glass was a viscoelastic material (together with earlier work on stress calculations, relaxation phenomena, and creep in viscoelastic bodies [34] in 1959, [35] in 1960, and [36] in 1963). Later, ref. [37] confirmed through experimental research that glass is just such a material. This assumption was still a certain level of simplification, but it allowed not only the prediction of residual stresses, but also a more accurate demonstration of what happens in the vicinity of the transition temperature range and development of transient stresses during cooling.

The thermoelastic model allowed us to describe the time scale of stress relaxation versus temperature change. Additionally, the assumption that glass is a thermorheologically simple material means that the stress change time scale can be stretched or compressed as a function of temperature. The time measured according to this temperature-adjusted scale is called the “reduced time”. In addition, an equation that describes this modified time scale has been proposed and is called the shift function [38].

In 1969, ref. [29] proposed a modified numerical approach to the theory proposed by [33] and this allowed the numerical results to be more accurate and align better with the experimental results. This publication further demonstrates that this model is not accurate for cooling from lower temperatures and that further research needs to take into account structural effects. This publication was followed by another milestone paper by [39] in 1970 that proposed a combination of stress relaxation and structural relaxation. This particular work was mainly devoted to the tempering of glass and an attempt to describe the formation of residual stresses. Then, it was demonstrated that 40% of the residual stresses within the glass come from structural changes originating in the transition temperature range (volume and viscosity change).

One of the most groundbreaking publications was [40] from 1971, which proposed a multiparametric model to accurately describe structural relaxation. According to this publication, the greatest challenge was to capture the nonlinearity of the structural relaxation, which is caused by structural changes within the glass that affect the viscosity. The change in viscosity then affects the relaxation rate.

A first attempt to link the current state of the glass structure with viscosity was made in a publication by [41]. The Tool model at the time assumed the creation of a fictive temperature as a measure of the current structural state of the glass and described viscosity as a function between the actual temperature and the fictive temperature. Ref. [40] stated that this model is adequate for one particular case because it assumes only one simple exponential relaxation mechanism consisting of only one relaxation time. In fact, glass exhibits a more complex relaxation phenomenon, resulting from having what is called the “memory effect”, which is the influence of previous thermal treatments on future behavior at elevated temperatures. This has been well described by [42] in 1969. The topic was also covered in an earlier publication by [43] from 1956, which devoted much attention to the study of the thermal history phenomenon of a given glass and showed a large influence on the properties of the material.

The subsequent work by [44] from 1978 further developed the model and demonstrated its application to tempered glass modeling and confirmed that the previously proposed approach can accurately predict residual stresses for both tempered and heat-treated glass. It was reduced (to 24%), but also confirmed previous assumptions that structural relaxation plays an important role in the heat treatment process, and should not be neglected and should be included in models.

In 1987, ref. [45] developed a modified equation proposed by [40] in 1971 that allowed for a simpler and more efficient calculation of the fictive temperature. This equation was also more stable since the step size during the calculation was independent of the relaxation time, which varies exponentially with temperature.

It is worth mentioning that most of these recent models have been developed and subsequently refined to model the stress distribution resulting from the toughening of glass, i.e., the rapid cooling that causes compressive stresses to occur on the surface of the glass and tensile stresses to occur within the glass. A detailed analysis of the distribution of these stresses is presented in [46]. The result of this combination is an increase in the mechanical strength of the glass due to the fact that glass shows a higher compressive strength than a higher tensile strength. The process of glass toughening is quite complex and differs for glass sheets of different thicknesses, geometry, number of holes, and how the process itself is carried out, i.e., the temperature to which the glass is heated, time of heat treatment, and the rate of its cooling. The work of [47] devoted considerable attention to toughened glass and the theory of toughening. One of the first publications that summarized the knowledge of time in order to approach numerical simulations of glass tempering was [48,49]. The latter work has, for the first time, comprehensively brought together existing knowledge on glass models and implemented it through numerical computations in the MARC software.

As a result of the increasing use of more complex flat geometries and point fixings, the tempering process itself has faced new challenges. Any change in geometry, for example sharp corners or chamfering of holes for point fixings, impairs the uniform cooling process, which has an impact on the final stress distribution in the glass [50]. Many research papers have been written on the subject, with the first notable publication appearing in the late 1990s prepared by [51]. The work focused on the analysis of the transient and residual stresses of a thin glass sheet in the inner zone and at the edge. The results were then compared with experimental studies, which confirmed the convergence of the analyses. In addition, the authors conducted an analysis of the sensitivity of the model to various material parameters required by the theoretical model. An additional important element of this publication was the determination of the thermal transfer coefficient, which was a previously unknown parameter (strictly dependent on the quenching process). This parameter describes forced convection during the cooling of glass sheets during the tempering process.

#### Nielsen [52] Work

One of the most influential works of recent years is that of [53], who proposed a constitutive model for glass and comprehensively described the implementation of an algorithm for 3D simulation in a finite element method software ABAQUS [54] using on UMAT subroutine. The model was based on two assumptions: that the glass behaves as a thermorheologically simple material and that it takes into account the structural relaxation, which describes the variation of the glass density with the cooling rate. This numerical model was validated through a series of experiments that confirmed the accuracy and correctness of the model.

The current model to describe the behavior of glass at elevated temperatures can be broken down into three components: temperature history, structural relaxation, and temperature-dependent viscoelasticity. Assuming that the temperature history is known, we can focus on the two aspects, i.e., temperature-dependent viscoelascity and thermorheologically simple behavior; and structural relaxation.

The principal constitutive equation is written as a hereditary integral. This type of integral obtained from the transformation of a generalized Maxwell model allows the description of stresses without the need to build models consisting of springs or dampers and all what is required is knowledge of the relaxation function (described below as Prony series, which is widely used to describe experimental results of viscoelastic behavior, e.g., for laminated glass and polymeric various interlayers presented in [55]). In the basic model of viscoelasticity, the input information, i.e., strain (creep) or stress (relaxation), is constant; when these values are variable, the Boltzmann superposition principle applies. The main equation is described as follows:

(1) $$\sigma_{i}{}_{j}\left(t\right)=2\int_{0}^{t}G\left(\xi -\xi^{\prime }\right)\frac{de_{i}{}_{j}\left(t^{\prime }\right)}{dt^{\prime }}dt^{\prime }+\delta_{i}{}_{j}\int_{0}^{t}K\left(\xi -\xi^{\prime }\right)\frac{\varepsilon_{i}{}_{j}\left(t^{\prime }\right)}{dt^{\prime }}dt^{\prime }$$

where $\sigma_{i}{}_{j}$ represents the stress tensor, *G* and *K* are the time-dependent shear and bulk moduli, respectively, ξ is the scaled time (or the shifted time), $e_{i}{}_{j}$ is the deviatoric strain tensor, $\varepsilon_{i}{}_{j}$ is the trace of the strain tensor, *t* is the time and $\delta_{i}{}_{j}$ is the Kronecker delta.

- Temperature-dependent viscoelasticity and thermorheologically simple behavior.

Both relaxation moduli can be written as series of exponential series called the Prony series:

(2) $$G\left(t\right)=\sum_{n=1}^{N_{G}}g_{n}exp\left(-\frac{1}{\lambda_{n}^{g}}\right)$$

(3) $$K\left(t\right)=\sum_{n=1}^{N_{K}}k_{n}exp\left(-\frac{1}{\lambda_{n}^{k}}\right)$$

This is a representation of a generalized Maxwell model, where each Maxwell element is composed of a spring with stiffness $g_{n}$/$k_{n}$ and a dashpot with relaxation time $\lambda_{n}^{g}$/$\lambda_{n}^{k}$.

Scaled time (or shifted time), ξ, is a specific property of viscoelastic materials that expose thermorheologically simple behavior. With a simple scaling of time *t*, the variation of relaxation moduli at different temperatures can be described only by knowing the properties of the material at the base temperature. The equation is described as:

(4) $$\xi =\int_{0}^{t}\phi \left(T\left(t^{\prime }\right)\right)dt^{\prime }$$

where $t^{\prime }$ is the running (time) parameter, and ϕ is the temperature-dependent time-scaling function. There are various shift functions available that describe different types of materials. One of the functions describing glass and its viscosity changes can be based on Arrhenius law and derived as [52,56]:

(5) $$\ln\phi \left(T\right)=\frac{H}{R_{g}}\left(\frac{1}{T_{B}}-\frac{1}{T}\right)$$

where *H* is the activation energy for glass, $R_{g}$ is the universal gas constant, *T* is the current temperature and $T_{B}$ is the base temperature for which the master relaxation curve is specified. Another frequently used shift function is the Williams-Landell-Ferry (WLF) equation proposed in [57].

(6) $$\log A_{T}=\frac{C_{1}^{g}\left(\theta -\theta_{g}\right)}{C_{2}^{g}+\left(\theta -\theta_{g}\right)}$$

where $A_{T}$ is the horizontal shift factor, $C_{1}^{g}$ and $C_{2}^{g}$ are constants, and $\theta_{g}$ is the glass transition temperature.

- Structural relaxation.

The second part of the model is the occurrence of so-called structural relaxation. The changing structure of the glass as it cools causes changes in viscosity, which in turn affects the rate of relaxation. As mentioned above, ref. [41] suggested that a fictive temperature $T_{f}$ could be introduced to capture the microstructure of the glass. The equation is described as follows:

(7) $$T_{f}\left(t\right)=T\left(t\right)-\int_{0}^{t}M\left(\xi \left(t,T_{f}\right)-\xi^{\prime }\left(t,T_{f}\right)\right)\frac{\partial T(t^{\prime })}{\partial t^{\prime }}dt^{\prime }$$

where *M* is the response function for certain properties (here volume), ξ is the scaled time, and $T_{f}$ is the fictive temperature. Then, the result of this equation $T_{f}$ goes back to the scaled-time equation (Equation (6)), which now looks as follows:

(8) $$\ln\phi \left(T\right)=\frac{H_{g}}{R_{g}}\left(\frac{1}{T_{B}}-\frac{1}{T}\right)+\frac{H_{s}}{R_{g}}\left(\frac{1}{T_{B}}-\frac{1}{T_{f}}\right)$$

where $H_{g}$ is the activation energy related to the temperature part and $H_{s}$ is the activation energy related to the atomic structure of the glass.

Once the fictional temperature has been calculated, we can use it to determine the thermal deformation through the following equation:

(9) $$\Delta \varepsilon_{ij}^{th}=\delta_{ij}\Delta \varepsilon_{th}=\delta_{ij}\left(\alpha_{g}\Delta T+\left(\alpha_{l}-\alpha_{g}\right)\Delta T_{f}\right)$$

where $\alpha_{l}$ and $\alpha_{g}$ are isotropic liquid and solid state thermal expansion coefficients, respectively. This equation accurately describes how the change in fictional temperature replicates the change in structure with temperature variations.

- Liquid state ($T_{f}=T$)—$\alpha_{g}$ is not a part of the equation anymore;
- Supercooled liquid state ($T<T_{f}$)—in this case by cooling, $T_{f}$ lags behind the real temperature;
- Glass state ($\Delta T_{f}=0$)—$\alpha_{l}$ is not part of equation anymore.

The key parameters of the above model are summarized in Table 1, together with an assessment of their impact on the model, together with units in the SI system.

### 3.2. Experimental Study of Key Parameters

Parallel to the development of mathematical models, knowledge of glass as a unique material and its physical parameters has also increased. Although glass has been used for many millennia, intensive studies on psychical parameters started in the first half of the XIX century by [58] with rapid developments in the latter half. This was dictated by the development of research methods and the general progress of science. Today, most of the parameters of SLS glass material used for research purposes come from a limited amount of work that has been worked on in the future [5,59,60]. The values of the parameters listed in these publications are used throughout the business as a basis and are frequently quoted. The architectural glass production process continues to improve and with it the quality of the end product. As the chemical composition of glass has a direct impact on mechanical properties, it is important to keep this information up-to-date to better model its behavior, particularly at elevated temperatures.

The direct modeling behavior of glass is influenced by many of its material characteristics, where some have a greater influence, and others have a lesser influence on the production and therefore modeling process. The most important aspect is that the properties of SLS glass can vary significantly with temperature changes. It is by manipulating these properties that it is possible to produce tempered glass, which has significantly improved mechanical strength and resistance to thermal shock, which is more often required; for example, also for various combinations of installations [61]. Based on a sensitivity analysis for the formation of residual stresses performed by [51], the most influential parameters are the glass transition temperature $T_{g}$, the solid/liquid thermal expansion coefficient ($\alpha_{L}$/$\alpha_{V}$), Young modulus (*E*) and the specific heat capacity $C_{p}$. The glass transition temperature $T_{g}$ is strongly dependent on the chemical composition of the glass, and therefore small changes can lead to significant discrepancies in residual stress values. The annealed glass can be considered an isotropic material (in contrast to thermally strengthened glass, which exhibits anisotropy) and chemically homogeneous, which further allows one to assume that the thermal expansion of the liquid is three times higher than the thermal expansion of the solid, that is, $\alpha_{V}$ 3$\alpha_{L}$ [47]. The Young modulus decreases in value as the temperature increases, and will be discussed in further detail here. Specific heat capacity $C_{p}$ also changes its value markedly with increasing temperature, and its changes are used to determine the glass transition temperature.

Table 2 presents summarized information on the material parameters of the available publications. The main conclusion that was observed is that information is based on older publications, and with the rapid development of glass technology, it was decided to investigate a few key parameters based on two commercial SLS glass products currently used in the building industry.

**Figure 3:**
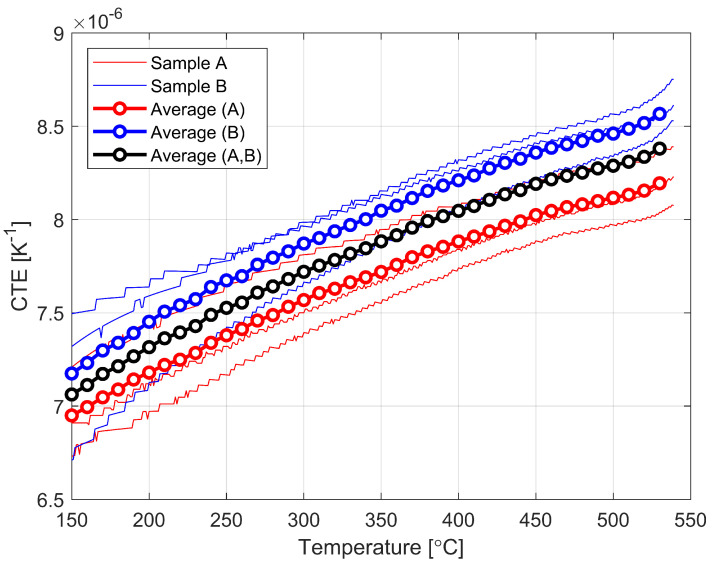
CTE test results for two sets of samples.

Figure 3 shows the results collected for the CTE test of the six glass samples and the average values. The two sets each of 3 samples of the same type of glass from two different manufacturers were tested and described as Sample A and Sample B, as mentioned earlier. The vertical axis is the value of the thermal expansion coefficient and the horizontal axis is the temperature. The graph is shown in the range from 423 K to 813 K. The solid lines represent the results for individual samples, while the lines with points represent the averaged values, with the black line marking the averaged value for all samples. As a result of analyzing the values obtained from both studies, the most prominent observation is that the obtained values are slightly lower than those available in the literature. As can be observed, as the temperature increases, the CTE increases in an approximately linear manner. Values between individual samples can vary by up to 10%. An approximate function ($R^{2}$ = 0.9995) to describe the solid thermal expansion coefficient can be described as follows:

(10) $$\alpha_{S}=-4\times 10^{-12}T^{2}+6\times 10^{-9}T+6\times 10^{-6}$$

The values obtained are lower than those reported in the literature (see Table 2). For a similar temperature range (approximately 823 K), the average value of the coefficient is 8.4 × 10^−6^ K. However, it should be noted at this point that the values available in the literature do not provide information on the chemical composition of the glass tested at the time. This, in turn, can have a significant impact on material parameters. The glass samples used in the experiment are a standard product available on the construction market, and the main objective is to update the available information. A precise comparison of the impact of changes in chemical composition is beyond the scope of this article.

**Figure 4:**
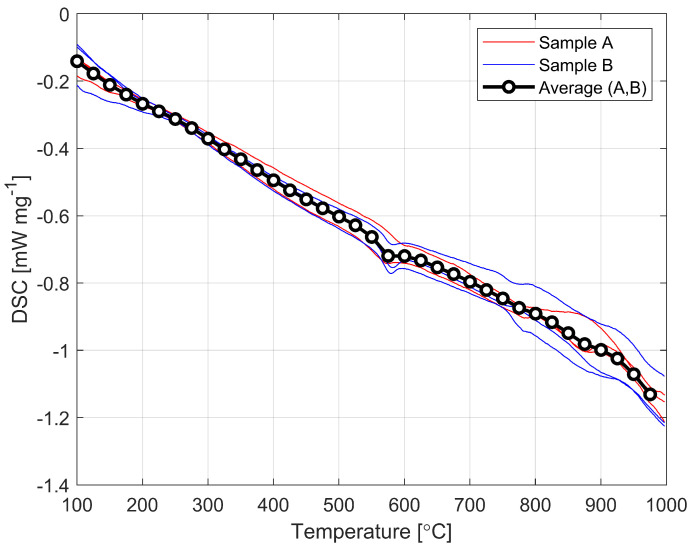
DSC graph for two sets of samples.

**Figure 5:**
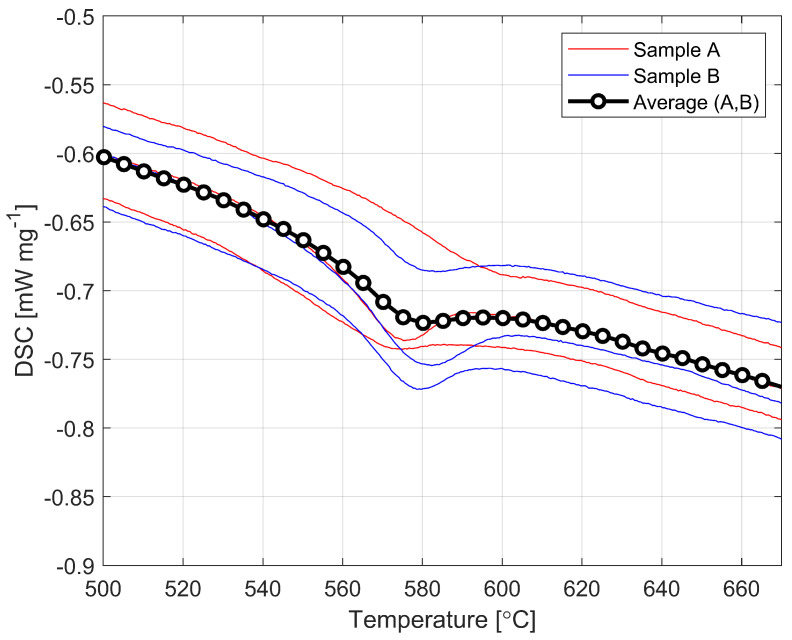
DSC graph in glass transition range.

Figure 4 shows the graph showing the results of the DSC testing exactly the same sets of samples of which the test results were shown on Figure 3. These curves are automatically generated in the instrument and from these the glass transition temperature is calculated in the range when we start to observe changes in the value of specific heat capacity (shown in Figure 5).

The values obtained at which the transition occurs are 825 K and 820 K, respectively. Averaging all measurements, we obtain a transition temperature calculated at $T_{g}$ = 823 K. This is approximately 32 K (4%) lower than the average value of the transition temperature found in the available literature. The magnitude of this difference cannot be ignored and the lower values obtained from the tests would have an impact on the modeling results.

**Figure 6:**
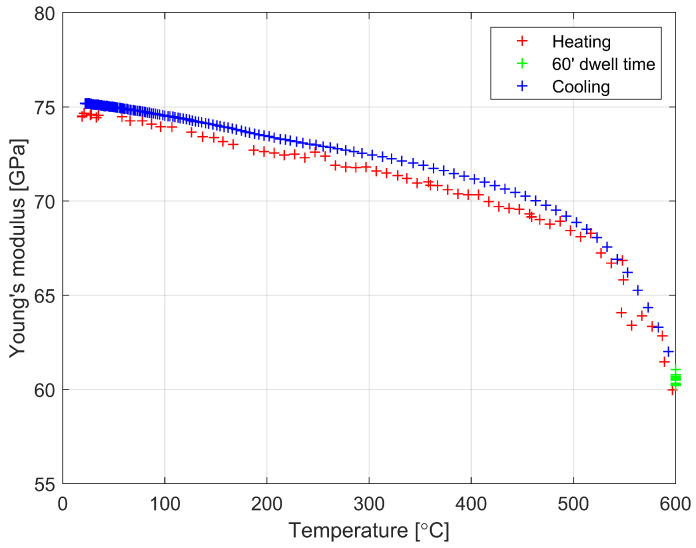
Dependencies of Young’s modulus versus temperature during the heating and cooling of soda lime glass with 60 min of dwell time at 873 K.

Figure 6 shows the changes in Young’s modulus in the temperature range from 295 K to 873 K. Above temperatures of 773 K, a marked acceleration of the decrease in Young’s modulus is observed. Young’s modulus on heating to 773 K decreases from 74 GPa (*G* = 30.5 GPa, υ = 0.220) to 68 GPa. Most likely, it is related to the transition of the material from the elastic to viscoelastic state.

**Figure 7:**
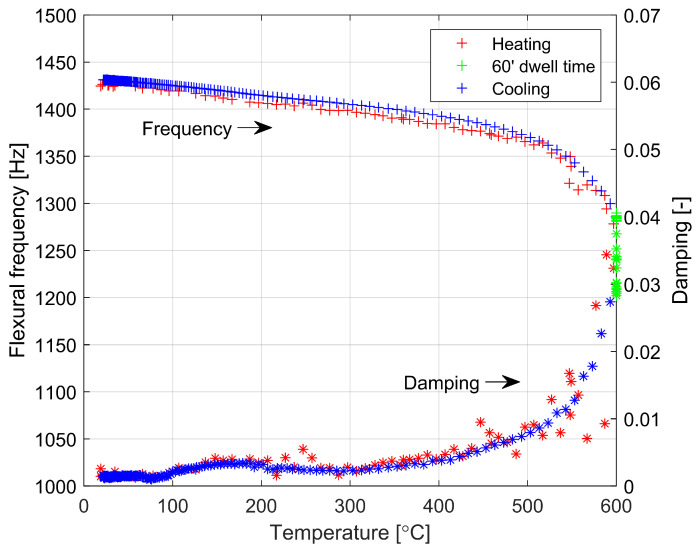
Dependencies of flexural frequency and damping versus temperature during the heating and cooling of soda lime glass with 60 min of dwell time at 873 K.

**Figure 8:**
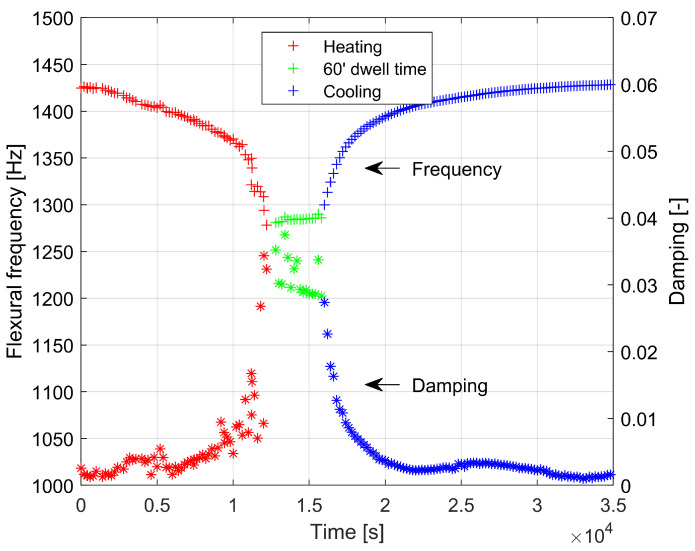
Dependencies of flexural frequency and damping versus time during heating and cooling of soda lime glass with 60 min of dwell time at 873 K.

This is accompanied by a marked increase in internal friction (damping), which is shown in Figure 7. If the material becomes viscoelastic above a temperature of about 773 K, the calculation of Young’s modulus from resonance frequencies loses its sense of the theory of material elasticity. For this reason, in Figure 7 and Figure 8 the results are presented as a function of the frequency of the flexural vibrations versus temperature and time, respectively. At temperatures above 773 K, a significant increase in internal friction (damping) is observed, which confirms the transition of the material to a viscoelastic state. This transition is blurred over a wider temperature range. An interesting observation is also a slight increase in internal friction (damping) in the material in the temperature range of 363 K to 543 K, both during heating and cooling. This increase is not accompanied by an observable effect on the temperature dependence of Young’s modulus. This observation should be explained by changes in the material at the atomic scale occurring in this temperature range and will require clarification in the course of further research.

## 4. Conclusions

Glass modeling, in particular, in a wide temperature range, is a demanding task but simultaneously is a key effort to improve the production of bent glass. In particular, advaced glass consitituve modelling, and the ability to model the specific geometry of the glass and the operating parameters of the furnace, make it possible to accurately estimate residual stresses. This is fundamental in order to be able to create a safe product for the end customer. As the glass manufacturing process evolves, so do the physical parameters of the glass. Therefore, it is important that the key material parameters used in the model are up-to-date and represent the glass currently in use.

The main objective of the research was to analyse the available literature with respect to the glass bending process, identify the most important parameters of this process and then test these parameters for the glass currently used in the construction industry. The study of literature has found that the change of the coefficient of linear expansion (CTE), Young’s modulus with respect to temperature and the glass transition temperature are the most important physical parameters of glass needed for the correct simulation of glass modeling. The further experimental research provided new, updated values of those parameters, and the slight differences in the values obtained suggest that the composition of glass currently used in construction has changed since the previously published results in the 1990s. The results of this research will provide new and more accurate data that will serve to further develop the modelling of bent glass and the manufacturing process itself.

Future research will be primarily focused on carrying out numerical simulations using the parameters presented and validating the results by bending real glass samples in a furnace.

## Figures and Tables

**Table 1:** Summary of model parameters and impacts acc. to Daudeville and Carre [51].

	Parameter	Parameter Description	Unit	Impact on the Model
1	$T_{ref}$	Reference temperature	K	High
2	$\alpha_{l}$	Liquid glass thermal expansion coeff.	10${}^{-6}$ K	High
3	$g_{n}$, $k_{n}$	Shear relaxation and bulk relaxation moduli	Pa	High
4	$\lambda_{n}^{g}$, $\lambda_{n}^{k}$	Relaxation times	s	High
5	$\lambda_{th}$	Thermal conductivity	W m${}^{-1}$ K${}^{-1}$	Medium
6	$C_{p}$	Specific heat capacity	J kg${}^{-1}$ K${}^{-1}$	Low
7	$\alpha_{s}$	Solid glass thermal expansion coeff.	10${}^{-6}$ K${}^{-1}$	Low
8	ρ	Density	kg/m${}^{3}$	N/A
9	*H*	Activation energy (total)	J/mol	N/A
10	$R_{g}$	Ideal Gas constant	J/mol	Physical constant
11	*h*	Forced convection constant	W m${}^{-2}$ K${}^{-1}$	Environment variable
12	$T_{init}$	Initial Temperature	K	Environment variable
13	$T_{\infty }$	Ambient Temperature	K	Environment variable

**Table 2:** Review summary of selected parameters.

Paper	Date	$T_{g}$	$\alpha_{L}\left(\alpha_{V}\right)$	*E*	$C_{p}$
Lee et al. [33]	1965	811	-	-	-
Narayanaswamy and Gardon [29]	1969	811	-	7.58	-
Narayanaswamy and Gardon [29]	1970	811	-	-	-
Ohlberg and Woo [62]	1974	-	9.5 (24.5)	-	-
Soules et al. [63]	1987	746	9.2 (18.4)	-	-
Pilette and Taylor [64]	1988	-	8.0	7.0	-
Plumb [65]	1989	823	-	-	-
Carré and Daudeville [49]	1996	869	9.1 (27.1)	7.2	-
Daudeville and Carre [51]	1998	869	9.1 (25.1)	7.0	-
Carré and Daudeville [59]	1999	864	9.0 (25.0)	7.0	-
Laufs and Sedlacek [48]	1999	853	-	-	1000 (at 853 K)
Pelletier et al. [66]	2000	813	9.8	6.2	-
Shelby [1]	2005	823–853	9.5	7.0–7.5	-
Nielsen [52]	2009	869	9.1 (25.1)	7.0	-

## Data Availability

Not applicable.
